# Tonic Endocannabinoid Levels Modulate Retinal Signaling

**DOI:** 10.3390/ijerph191912460

**Published:** 2022-09-30

**Authors:** Charles F. Yates, Jin Y. Huang, Dario A. Protti

**Affiliations:** 1 School of Medical Sciences (Neuroscience), The University of Sydney, Sydney, NSW 2006, Australia; 2 Department of Neurosurgery, Royal Brisbane and Women’s Hospital, Brisbane, QLD 4029, Australia; 3 School of Medical Sciences (Education Innovation), The University of Sydney, Sydney, NSW 2006, Australia

**Keywords:** endocannabinoids, retinal ganglion cells, light-responses, URB597, fatty acid amide hydrolase

## Abstract

The endocannabinoid (eCB) system is critically involved in the modulation of synaptic transmission in the central nervous system, playing an important role in the control of emotional responses, neurodevelopment and synaptic plasticity among other functions. The eCB system is also present in the retina, with studies indicating changes in function after application of cannabinoid receptor agonists, antagonists and in knockout models. Whether eCBs are tonically released in the retina and their physiological functions is, however, still unknown. We investigated the role of the eCB system in the modulation of response strength of retinal ganglion cells (RGCs) to light stimulation, their receptive field organization, contrast sensitivity and excitability properties by performing whole-cell patch-clamp recordings in mouse RGCs before and after bath application of URB597, an inhibitor of the enzyme that degrades the eCB anandamide. Our results show that URB597 application leads to a reduction in the strength of synaptic inputs onto RGCs but paradoxically increases RGC excitability. In addition, URB597 was shown to modulate receptive field organization and contrast sensitivity of RGCs. We conclude that tonically released eCBs modulate retinal signaling by acting on traditional cannabinoid receptors (CB1R/CB2R) as well as on non-cannabinoid receptor targets. Thus, a thorough understanding of the effects of drugs that alter the endogenous cannabinoid levels and of exogenous cannabinoids is necessary to fully comprehend the impact of their medical as well as recreational use on vision.

## 1. Introduction

Cannabis has been used by humans for several thousand years for recreational and spiritual purposes due to its psychoactive properties, as well as therapeutically to treat many medical conditions ranging from pain to nausea. While cannabis is the most widely used illegal drug in the world, its use for medical and/or recreational purposes has recently been approved in several countries and most states in the USA, with heavy cannabis use consistently increasing in the last 15 years [1]. A large body of evidence has accumulated supporting the use of different phytocannabinoids in the treatment of cancer, multiple sclerosis, epilepsy, neurodegenerative and inflammatory disorders among other diseases [2,3,4]. Despite the therapeutic potential of cannabis, its psychotropic effects, for which it is sought after and consumed by recreational users, raise numerous safety concerns that include psychological changes as well as cognitive impairment such as deficits in attention, decreased reaction time and working memory as well as perceptual anomalies. Cannabis has been identified as the second most common substance involved in traffic accidents, including fatalities, due to impaired driving [5].

The main psychoactive substance in marijuana is ∆9-tetrahydrocannabinol (THC), which binds to cannabinoid receptors that are physiologically activated mainly by the endogenous cannabinoids (eCBs) anandamide (AEA) and 2-acylglycerol (2-AG). The eCBs, their respective receptors and the synthesizing and degrading enzymes comprise the endocannabinoid system. eCBs have been identified as key modulators of synaptic function in many regions of the central nervous system (CNS), including the hippocampus, basal ganglia, cerebellum, visual cortex and the retina among other areas [6,7,8].

eCBs and type 1 cannabinoid receptors (CB1R) have been localized to the processes of most retinal cells in the inner and outer plexiform layers (IPL and OPL) of the monkey, mouse, human and other species [9,10,11,12]. In addition, CB1R and fatty acid amide hydrolase (FAAH), the degrading enzyme of AEA, were found in all retinal cells from early developmental stages, in rat and vervet monkey models [13,14,15,16]. We have previously shown that synthetic cannabinoids regulate the strength of light responses in ON α-RGCs (retinal ganglion cells) as well as the spatial organization of their receptive fields [17], suggesting that exogenous cannabinoids affect vision at the very first stage of visual processing. Moreover, manipulation of the cannabinoid system by activating CB1R receptors or inhibiting FAAH degradation showed evidence of an active endocannabinoid system in primate retina by recording non-specific mass electroretinographic responses. It is unknown, however, whether or not modulation of the endocannabinoid retinal system impacts on the output signals from the retina to higher visual areas in the brain. In this study, we investigated the effects of URB597, an inhibitor of the degrading enzyme of anandamide on the response strength, receptive field properties and contrast response of RGCs to address the gap in knowledge of the effects of cannabis use on the visual system.

## 2. Materials and Methods

All procedures were approved by the Animal Ethics Committee of The University of Sydney (protocol #: 661) and followed the guidelines for animal experiments as set out by the Australian Code of Practice for Care and Use of Animals for Scientific Purposes, and the National Health and Medical Research Council of Australia.

### 2.1. Tissue Preparation

Adult mice (C57Bl/6J; >4 weeks, 13 animals) of either sex were dark-adapted for at least 2 h before anaesthesia with isofluorane, followed by euthanasia by cervical dislocation under dim red light. Both eyes were enucleated and dissected in carboxygenated Ames medium in the dark under infrared light using infrared night viewers (Find-R-Scope, FJW optical systems) to maintain retinal dark-adaptation. The cornea, iris, and vitreous were successively removed; the eyecup was cut into halves and the hemiretinas were detached from the sclera. A hemiretina was then mounted in a recording chamber photoreceptor side down and transferred to the stage of an upright microscope (Axioskop 40, Zeiss). Retinal tissue was visualised on an LCD monitor using a CCD camera under infrared illumination (>900 nm) using differential interference contrast (DIC) optics. Cells in the ganglion cell layer were exposed by tearing a small hole in the inner limiting membrane with a glass pipette to gain access to the cell bodies of ganglion cells [18].

### 2.2. Recordings

Whole-cell patch-clamp recordings were conducted using borosilicate glass pipettes with a resistance of 6–10 MΩ. High resistance seals (>1 GΩ) were made on the soma of large neurons (>18 µm diameter) in the ganglion cell layer. Recordings were made in both current–clamp and voltage–clamp configurations, with holding membrane potential set at −60 mV. Patch electrodes were filled with an intracellular solution containing (in mM): K-gluconate: 140, MgCl_2_: 4.6, EGTA: 10, HEPES: 10, ATP-Na: 4 and GTP-Na: 0.4, 20 creatine phosphate, and 250 U/mL creatine phosphokinase. Lucifer yellow (2%) was added to the intracellular solutions for cell identification. Ames medium (36 °C) was continuously perfused at 3 mL/min. All chemicals and drugs were obtained from Sigma except for Ames that was purchased from US Biological. Responses were recorded using an EPC9 amplifier and Pulse 8.67 software (HEKA Elektronik, Reutlingen, Germany).

### 2.3. Visual Stimulation and Recording Protocols

Retinal tissue was continuously exposed to a background of 0.025 cd/m^2^ mean luminance (mesopic conditions). Visual stimuli were focused on the photoreceptor layer of the retina through the microscope optics using a miniature monochromatic white OLED projector (800 × 600 pixels, SVGA+XL Rev2, eMagin) at 60 Hz. Spontaneous spike rate was determined by a 2 min continuous recording on background luminance. Stimuli consisted of bright uniform circular spots of varying diameters (12 different sizes between 50 μm and 1100 μm). Achromatic stimuli were presented for 500 ms on the background luminance at an intensity of 0.25 cd/m^2^ (83% Michelson contrast). We define optimal spot as the spot size that produced the strongest response. Optimal spot size was used to study responses to different contrasts. Spot size protocols were run twice for the purpose of averaging. Light-evoked responses were recorded before and 5 min after bath application of the FAAH inhibitor URB597 (1 µM); stock solutions of URB597 were prepared in DMSO (DMSO concentration in Ames solution was maintained at <0.1%). See Supplementary Figure S1 for the configuration of our recording setup.

Stimuli were generated using the visual stimulus generating software EXPO (P. Lennie, University of Rochester, Rochester, NY, USA).

### 2.4. Data Analysis

Data analyses were conducted using custom written routines in Igor Pro (Wavemetrics, Lake Oswego, OR, USA).

Stimulus-evoked responses were characterised by quantifying the number of action potentials as well as the amplitude of the light-evoked postsynaptic potential (LE-PSP) following light stimulation. Action potentials were detected by using an off-line routine to locate the maxima by calculating the smooth first and second derivative of the voltage signal and comparing it to a threshold typically set between −35 mV and −30 mV. Spontaneous spike frequency was subtracted from stimulus-evoked spike responses. To measure the amplitude of LE-PSPs, spikes were removed by linear interpolation of the membrane potential signal 3–4 ms before each spike and 8–10 ms after each spike. Mean amplitude of LE-PSP was measured as the difference between a baseline averaging 200 ms before stimulus delivery and the average membrane potential across 50–100 ms following peak responses.

#### 2.4.1. Area–Response Function

The spatial organisation of receptive fields was analysed by measuring area–response functions from spike counts and the amplitude of LE-PSP visual-evoked responses. Spike output was quantified as a function of stimulus diameter. The maximum value from the area response function represents the ‘peak response’, which is an indicator of the receptive field centre size. A suppression index (*SI*) was calculated to estimate the degree of inhibition induced by stimulation of the receptive surround by using the following formula:

$$SI=\left(1-\left(R_{max}/R_{peak}\right)\right)\times 100$$

where *R_peak_* is the peak response and *R_max_* is the magnitude of the light-evoked response obtained using the largest spot size that stimulates both the centre and surround of the receptive field.

#### 2.4.2. Contrast Response Function

The amplitude of light-evoked post-synaptic potentials (LE-PSP) was used to analyse contrast sensitivity, due to low firing rates at lower contrasts, as this allowed for more accurate analysis. LE-PSP amplitude was quantified against contrast, which varied linearly from 0 to 83% in 10 steps, and the data were fitted with a Naka-Rushton function [19] using the following formula:s

*C*(*x*) = *R_Max_* × *C* × (*x^m^*/(*x^m^* + (*C_50_*)*^m^*))

as in [20] where *R_Max_* is maximal response, C is contrast, m is the slope of the curve and *C_50_* is the half saturation point (contrast at 50% of maximum LE-PSP amplitude). The *C_50_* value was obtained to compare contrast sensitivity in control conditions and in the presence of URB597.

### 2.5. Statistics

Values in the text and figures represent mean ± SEM (standard error of the mean). Statistical significance between two groups was calculated using a paired Wilcoxon signed-rank statistical-test unless otherwise stated. This non-parametric statistical analysis was chosen due to a non-normal distribution of data and variability between cells in terms of responses. The criterion for statistical significance was chosen to be *p* ≤ 0.05.

## 3. Results

**Figure 1:**
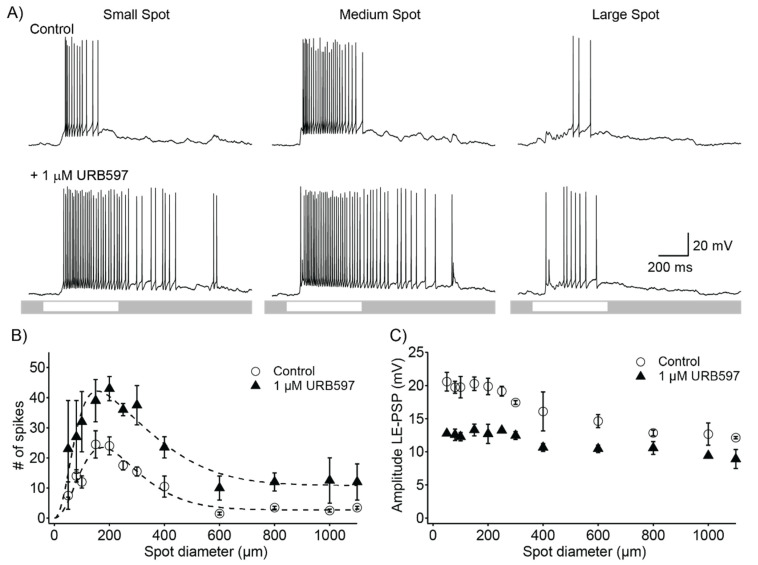
Effects of 1 µM URB597, a fatty acid amide hydrolase (FAAH) inhibitor, on light evoked responses of an ON retinal ganglion cell. (**A**) Responses from a representative cell to different sized spot stimuli. After addition of 1 µM URB597, response strength to all spot sizes is greatly increased. Small, medium, and large spot sizes were 50, 200, and 1100 µm, respectively. Lighter bars on grey background at the bottom of the traces indicate the timing of stimulus delivery and stimulus contrast. (**B**) Area–response function for spike count for the cell shown in (**A**) in control conditions (open circles) and in the presence of 1 µM URB597 (filled triangles). This cell showed the classical receptive field organization characteristic of many retinal ganglion cells, with an excitatory center and an inhibitory surround. Dashed lines show the best predictions of a difference-of-Gaussians of the receptive field. (# symbolizes number) (**C**) Area–response function for amplitude of light-evoked post-synaptic potential (LE-PSP) in control conditions (open circles) and in the presence of 1 µM URB597 (filled triangles). Symbols represent the mean and error bars are the standard error of the mean.

To investigate the potential role of the endocannabinoid system in modulating visual signal transmission at the first stage of image processing, we studied the effects of inhibiting the activity of the fatty acid amide hydrolase (FAAH), an eCB degrading enzyme, on light-evoked responses of retinal ganglion cells. Based on the well-described inhibitory effects of endo- and exo-cannabinoids on transmitter release in the central nervous system [21,22] and on our previous findings that an exogenous CB1R agonist reduces excitatory synaptic inputs to α-ON RGCs [17], we hypothesized that FAAH inhibition would lead to an increase in eCBs concentration and consequently cause a decrease in the strength of light-evoked responses in RGCs. Receptive fields of retinal ganglion cells were probed with circular spots of increasing diameter and their responses were quantified. Figure 1A shows representative light responses of an ON RGC. In this cell, responses to a small spot in control conditions produced a relatively weak response (Figure 1A, left top trace). Increasing spot size up to a larger diameter led to stronger responses (optimal size) and further increases in diameter led to weaker responses due to activation of the inhibitory surround (Figure 1A, middle and right top traces). Bath application of 1 µM URB597 produced an increase in the strength of light-evoked responses for all three spot sizes (Figure 1A, bottom traces). Figure 1B displays the area–response function for spike responses, for the same cell shown in Figure 1A, in control conditions and in the presence of URB597, revealing that FAAH inhibition induces an increase in firing rate for all sizes. The receptive field of this cell was well described by a difference-of-Gaussian function (DoG), consisting of an excitatory center and an inhibitory surround (dashed lines indicate fit to a DoG function). The direction of this modulatory effect of URB597 on retinal ganglion cell responses was opposite to its predicted effect. To gain insight into the mechanisms underlying the URB597 enhancing effect, we quantified its effect on the amplitude of the light-evoked post-synaptic potentials measured from responses in which spikes were filtered out. The area–response function of the LE-PSP reveals that URB597 reduced the magnitude of the LE-PSP for all spot sizes, opposite to its effect on spike response (Figure 1C).

**Figure 2:**
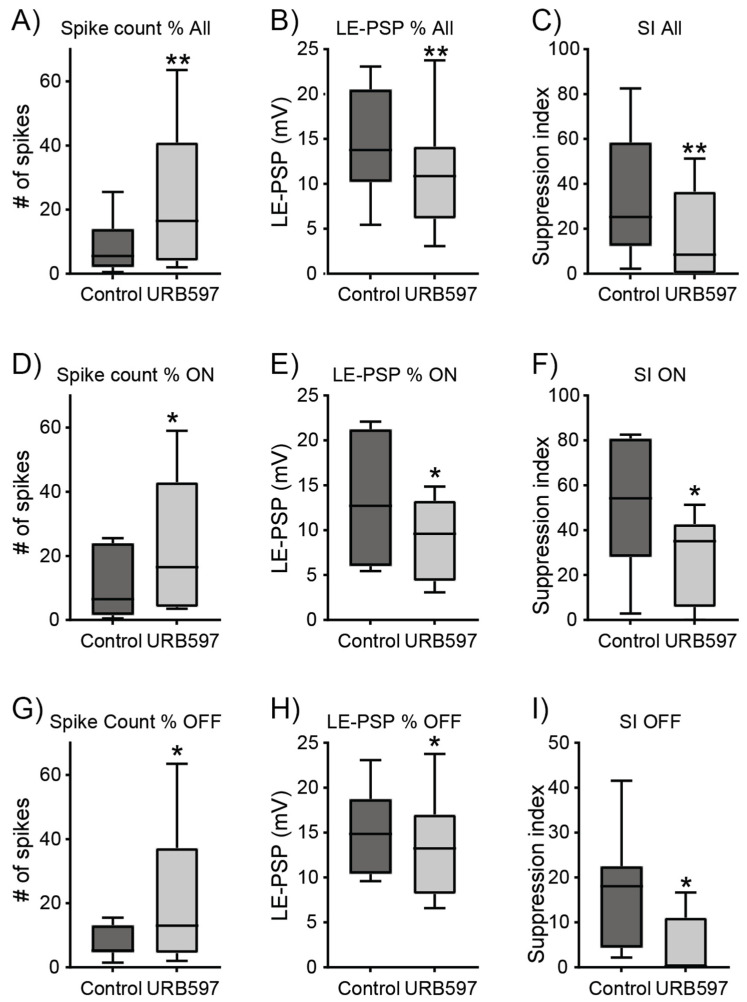
Effects of URB597 on spike response, amplitude of LE-PSP and suppression index of RGCs. (**A**) Spike count of the peak response in control conditions (dark grey) and in the presence of 1 µM URB597 (light grey) for all cells recorded. URB597 significantly increased spike count (*p* < 0.01, *n* = 15 cells from 13 mice). (**B**) Peak amplitude of LE-PSP was significantly reduced by 1 µM URB597 (*p* < 0.01, *n* = 15). (**C**) URB597 significantly reduced the suppression index of all cells (*p* < 0.01, *n* = 15). (**D**,**G**) Effect of 1 µM URB597 on spike count of the peak response of ON and OFF RGCs. URB597 significantly increased spike count (*p* < 0.05, *n* = 7 ON RGCs from 6 mice and 8 OFF RGCs from 7 mice). (**E**,**H**) Quantification of the effect of URB597 on peak amplitude of LE-PSP of ON (**E**) and OFF (**H**) RGCs showing that inhibition of the FAAH activity reduced the amplitude of the LE-PSP (*p* < 0.05, *n* = 7 ON and 8 OFF RGCs). (**F**,**I**) Suppression index was significantly reduced by URB597 in both ON and OFF cells (*p* < 0.05, *n* = 7 ON and 8 OFF RGCs). Whiskers indicate the smallest and largest values and horizontal lines indicates the median in each condition (# symbolizes number, * *p* < 0.05, ** *p* < 0.01).

Bath application of 1 µM URB597 produced a significant increase of 150% in the number of spikes of the peak response for all 15 ON and OFF RGCs recorded (Figure 2A; 9.1 ± 2.1 control vs. 22.7 ± 5.5 URB597, *p* < 0.05, *n* = 15). At odds with its effect on the spike response (Figure 2A), the mean amplitude of the peak depolarizing response was significantly reduced from 14.5 ± 1.6 mV in control conditions to 10.9 ± 1.5 mV in the presence of 1 µM URB597 (Figure 2B, *p* < 0.05, *n* = 15). To gain insight into the receptive field organization and the relative strength of its center and surround components, we estimated the suppression index. Inhibition of the FAAH activity by 1 µM URB597 significantly reduced the suppression index from 34.6 ± 7.5 in control conditions to 17.1 ± 5.1 (Figure 2C, *p* < 0.01, *n* = 15). Grouping cells by their response polarity as ON and OFF RGCs revealed that URB597 significantly enhanced the spike response in both cell types (Figure 2D,G, *p* < 0.05, *n* = 7 ON cells and 8 OFF cells), whilst it significantly reduced the peak amplitude of the light-evoked response and suppression index in both cell types (Figure 2E,F,H,I; *p* < 0.05, *n* = 7 ON cells and 8 OFF cells).

**Figure 3:**
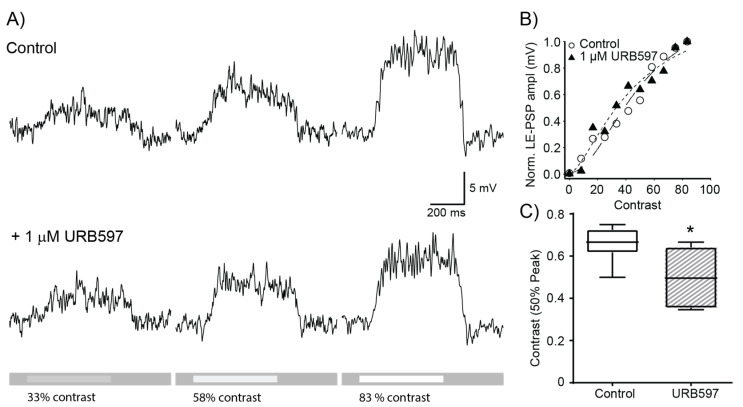
Effects of URB597 on contrast dependence of LE-PSP amplitude. (**A**) Light evoked postsynaptic potentials in control conditions (top traces) and in the presence of 1 µM URB597 (bottom traces) at 33, 58 and 83% stimulus contrast. Action potentials were filtered out for quantification of LE-PSP amplitude. Lighter bars on grey background at the bottom of the traces indicate the timing of stimulus delivery and stimulus contrast. (**B**) Contrast response curve for normalized peak amplitude of LE-PSP in control conditions and in the presence of URB597 for the same cell shown in (**A**). Lines denote the fit to Naka-Rushton equation (control: dashed line, URB597: dotted line). (**C**) Half-Saturation coefficients in control conditions and under 1 µM URB597. The half maximum response contrast was significantly reduced from 66 ± 4% to 50 ± 6% (*p* < 0.05, *n* = 6) in the presence of URB597 (* *p* < 0.05).

The observed reduction in suppression index indicates that URB597 reduces the strength of lateral inhibition, a critical mechanism involved in local contrast detection that contributes to enhanced perception of edges [23,24]. Therefore, we tested whether or not URB597 has a modulatory effect on the contrast sensitivity of RGCs by using optimal size spots of different contrast, ranging from 8.3% to 83%, to stimulate the receptive field center. To characterize the effects of URB597 on contrast sensitivity, we measured the amplitude of the LE-PSP as it provides a contrast-dependent response that varies in a graded manner and is therefore independent of the spike generation mechanism. Figure 3A shows representative recordings of an ON RGC in response to preferred contrast spots (33, 58 and 83% contrast, bright spots on grey background). Figure 3B shows the amplitude of the LE-PSP of the same cell, plotted against contrast. The contrast response function was fitted with the Naka-Rushton equation (dashed lines Figure 3B) and the characteristic parameters were extracted for assessing the effect of URB597. Inhibition of the FAAH activity by 1 µM URB597 significantly reduced the half saturation response from 66 ± 4% to 50 ± 6% (Figure 3C; *p* < 0.05, *n* = 6), suggesting that endocannabinoids increase the sensitivity to low contrast.

**Figure 4:**
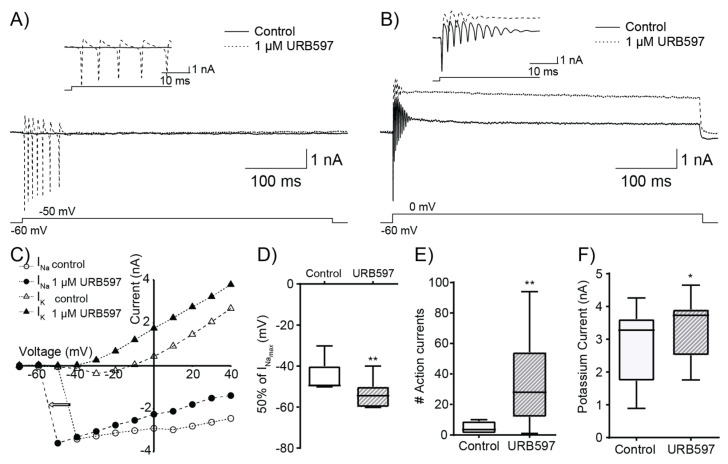
Application of the FAAH inhibitor URB597 alters excitability properties. (**A**) Representative sodium current elicited by a 500 ms depolarizing step from −60 mV to −50 mV in control conditions (solid line) and after bath application of 1 µM URB597 (dashed line). *Inset*: same currents at an expanded time scale. (**B**) Representative K^+^ currents elicited by a 500 ms depolarizing step from −60 mV to 0 mV in control conditions (solid line) and after bath application of 1 µM URB597 (dashed line). *Inset*: same currents at an expanded time scale. (**C**) Current–voltage Na^+^ and K^+^ curves in control (empty symbols) and after the application of URB597 (filled symbols) for the same cell. URB597 induced a leftward shift in the Na^+^ current (arrow) and an increase in the magnitude of the K^+^ current. (**D**) Membrane potential at which Na^+^ current reaches 50% of its peak amplitude. URB597 significantly reduced the voltage of the mid-point of peak Na^+^ current (*p* = 0.01, *n* = 12). (**E**) Effect of 1 µM URB597 on the number of inward Na^+^ currents evoked by depolarization to the lowest membrane potential that elicits Na^+^ currents in control conditions. URB597 induced a large, significant increase in the number of Na^+^ currents from 4.5 ± 1.2 events to 34.1 ± 9.5 events (*p* = 0.01, *n* = 12). (**F**) Summary of the effect of URB597 on peak K^+^ current amplitude. Mean K^+^ current amplitude was significantly increased from 2.84 ± 0.32 nA in control conditions to 3.44 ± 0.27 nA in the presence of 1 μM URB597 (*p* < 0.05, *n* = 12). * *p* < 0.05, ** *p* < 0.01.

The observed decrease in LE-PSP amplitude caused by URB597 is inconsistent with its enhancing effect on spiking output. This paradoxical effect suggests that eCBs may potentially act on postsynaptic targets in addition to their classical effect of reducing neurotransmitter release at the presynaptic level. To ascertain the effects of URB597 (1 μM) on membrane excitability properties, we conducted recordings in voltage–clamp mode to investigate the current-to-voltage relationships for Na^+^ and K^+^ currents. Membrane potential was held at −60 mV and stepped for 500 ms from −70 mV to +30 mV in +10 mV steps. Figure 4A illustrates representative voltage–clamp recordings in response to a depolarizing step from −60 mV to −50 mV in control conditions and in the presence of 1 µM of URB597. In control conditions, a 10 mV depolarizing step did not produce any changes in membrane current. Upon bath application of 1 µM URB597, a similar 10 mV step induced the repetitive activation of voltage-gated sodium currents. Figure 4B shows responses to depolarizing steps from −60 mV to 0 mV for the same cell in control conditions, and in the presence of URB597. Bath application of the FAAH inhibitor led to an increase in the magnitude of the outward potassium current. To characterise the effect of URB597 on cell excitability, we quantified three different properties of the current–voltage relationship. First, we quantified the degree of activation of Na^+^ currents by depolarization in control conditions and under URB597 by calculating the membrane potential at which 50% of the peak Na^+^ current occurred. Second, we quantified the number of Na^+^ events (action currents) observed at the lowest membrane potential that elicits sodium currents in control conditions. Finally, we measured the maximum amplitude of late-stage K^+^ currents (~450 ms after onset of the 500 ms depolarising steps).

Figure 4C illustrates representative current-to-voltage relationship curves obtained for an RGC. The amplitudes of Na^+^ and K^+^ currents are plotted against membrane potential. Peak Na^+^ current amplitude in control conditions was −3.4 nA, yielding a mid-point activation of −45 mV. After addition of URB597 (1 µM), the activation threshold for Na^+^ current was more negative, with a midpoint activation of −55 mV and peak amplitude was −3.6 nA. The leftward shift of the Na^+^ curve implies a change in threshold potential for Na^+^ currents. Figure 4D shows URB597 effect on the mid-point activation of the peak Na^+^ current, the mean potential at 50% of the peak Na^+^ current becoming significantly more negative from −44.3 ± 2.2 mV in control conditions to −53.9 ± 1.9 mV in the presence of 1 µM URB597 (*p* = 0.01, *n* = 12). Figure 4E shows the effect of URB597 on the number of events (sodium currents) at threshold testing voltage for all cells tested. These events are ‘action currents’ due to unclamped action potentials that originate in the axon hillock. Bath application of URB597 resulted in an increase in median event count from 3.5 in control conditions to 28 events in the presence of the FAAH inhibitor (*p* < 0.01, *n* = 11). This increase in events suggests changes in Na^+^ channel kinetic properties such as inactivation. Figure 4F illustrates the effect of URB597 on peak K^+^ current observed across all RGCs. Addition of 1 μM URB597 resulted in a significant increase in the median K^+^ current from 3.28 nA in control conditions to 3.73 nA in the presence of URB597 (*p* < 0.05, *n* = 12).

## 4. Discussion

Based on the known effects of the eCB system in the CNS, we hypothesized that enhancement of eCBs levels via inhibition of its degrading enzyme would decrease the strength of visual signals in retinal ganglion cells. Our results show that eCBs modulate the strength of visual signals in the mammalian retina but, paradoxically, their effect was to increase the spike rate of RGCs. Blocking the activity of the fatty acid amide hydroxylase had opposing effects on the amplitude of the light-evoked postsynaptic potentials and the ensuing spiking activity in RGCs. The decrease in LE-PSP is consistent with the classical presynaptic action of eCBs reducing neurotransmitter release whilst the increase in spiking activity appears to be mediated by a direct postsynaptic effect on RGCs. In addition, we observed that eCBs modify the contrast sensitivity of RGCs. These effects suggest a key role of eCBs on modulating retinal signal transmission and the information content of the signals transmitted to higher visual centers.

We have previously demonstrated that a CBR1 agonist reduced spiking activity of ON α-RGCs by decreasing the strength of excitatory inputs from bipolar cells [17]. In the present study, we observed that enhancement of eCB levels led to a similar reduction in the excitatory input into RGC, but also revealed a more complex interaction of the eCB system on different targets at the cellular level. Voltage–clamp recordings from RGCs were characterized by the presence of action currents due to unclamped action potentials arising from the axon, which prevented a thorough quantitative analysis of the activation and inactivation properties of voltage-gated sodium channels. URB597 shifted the activation curve of sodium channels to more negative potentials and increased the number of action currents as well as the magnitude of voltage-gated potassium currents, indicating that increases in eCBs levels enhance RGC excitability. It is well documented that in addition to activating CBRs, eCBs act on non-cannabinoid receptor targets that can account for some of the observed effects on RGC excitability. The transient receptor potential vanilloid type 1 (TRPV1) channel is expressed by a subset of RGCs in the mouse retina, and its activation by anandamide was shown to increase intracellular calcium concentration [25]. Calcium influx via TRPV1 receptors can activate calcineurin [26,27] leading to dephosphorylation of sodium channels and consequently reduce their time to inactivation [28]. Additionally, dephosphorylation of sodium channels due to raised intracellular calcium can shift the activation potential of sodium currents to more negative potentials via a protein kinase C-dependent mechanism [29].

Our findings highlighting the multiple sites of action of eCBs are consistent with other reports. A recent study found that activation of CBR1 selectively increases spontaneous GABAergic, but not glycinergic, inhibitory postsynaptic potentials in some types of bipolar cells [30]. This effect could contribute to the observed reduction in LE-PSP amplitude. CB1R activation was also shown to reduce visually evoked excitatory and inhibitory synaptic inputs and enhance excitability in amphibian RGCs [31]. These conflicting effects were due to presynaptic activation of CB1R reducing the strength of the synaptic inputs and a direct inhibitory effect on the Na^+^-K^+^-2Cl^−^ co-transporter 1 (NKCC1), leading to hyperpolarization and removal of inactivation of voltage-gated sodium channels [31].

Thus, it appears that the retinal cannabinoid system dynamically modulates the output of visual signals by regulating both transmitter release and neuronal excitability. An interesting observation by Ahluwalia et al. [32] was that anandamide might have differential effects at different concentrations: at low (nanomolar) concentrations the inhibitory CB1R mediated effects prevail while at higher (micromolar) concentrations, excitatory TRPV1 mediated effects would outweigh the CBR effects. Our results suggest that inhibition of endogenous anandamide degradation by FAAH might be sufficient for TRPV1 mediated effects to offset CBR effects. Although the magnitude of the change in AEA concentration following URB597 application is unknown, treatment with URB597 has been reported to double the concentration of AEA in the retina of young mice [33]. In addition, our preliminary experiments show that, similar to the effects of URB597, bath application of 15 µM AEA decreases the amplitude of LE-PSP, increases spiking output and shifts the activation curve of voltage-gated sodium channels to the left in mouse RGCs (unpublished data).

Jo et al. [25] and Ryskamp et al. [34] provided substantial evidence involving eCBs in a non-retrograde mode of action on TRPV1 channels. Based on these observations, a more thorough investigation of the role of TRPV1 channels in retinal function is necessary. Co-application of URB597 with capsazepine, a TRPV1 antagonist, could help elucidate if the URB597-induced shift in the input-output relationship is mediated by TRPV1. Interestingly, the enzyme calcineurin that is downstream of TRPV1 activation has been linked to RGC degeneration and death in animal models of glaucoma [35,36] and calcineurin inhibition has been suggested as a neuroprotective strategy for glaucoma [35]. Future experiments using URB597 in conjunction with FK506, a calcineurin inhibitor, could reveal whether calcineurin is a downstream target of eCB-mediated activation of TRPV1 channels.

The role of cannabinoid receptors in retinal signalling has also been explored by electroretinogram (ERG) recordings in CB1R and CB2R knockout mice. Cécyre et al. [37] showed a significant increase in the a-wave of the ERG, which reflects photoreceptor activity, in the CB2R knockout dark-adapted mouse compared to wild types but not in CB1R knockouts. However, the results were based on responses to flashes that are not optimal to stimulate the eCB system, which is regulated in an activity-dependent manner. The effects of cannabinoid agonists and antagonists have also been investigated in the ERG in the primate retina, where all components of the eCB system are present [38,39]. Significantly, intravitreal injection of antagonists of both CB1R and CB2R were shown to increase the b-wave component, which represents the degree of bipolar cell activity, of the scotopic and photopic response in the vervet monkey [38,39]. The ERG signal does not provide an accurate measurement of ganglion cell activity and it is therefore difficult to compare these reports to our results, nevertheless they highlight the key role of the eCB system in shaping the retinal output to higher visual centers.

A thorough understanding of the consequences of functional alterations in the eCB system is critical, as targeting CB receptors, inhibiting eCBs uptake and/or blocking their degradation are promising therapeutic approaches to treat eye diseases [40,41] as well as other CNS conditions such as Parkinson’s and Huntington’s disease [42,43], with several clinical trials underway (for review see [44]). The phytocannabinoids THC and cannabidiol (CBD) have been shown to be neuroprotective in experimental animal models of diabetic retinopathy and glaucoma [45,46,47]. CBD has also been shown to be neuroprotective in an NMDA-induced model of retinal neurotoxicity [46]. Similarly, a synthetic cannabinoid receptor antagonist was neuroprotective in an animal model of photoreceptor damage in vitro and in vivo [48]. Moreover, FAAH inhibition was found to have neuroprotective effects in rat models of high intraocular pressure (IOP)-induced ischemia [49] and optic nerve axotomy [33] by enhancing eCB levels that acted on both CB1Rs and TRPV1 channels. In addition to the aforementioned neuroprotective effects, Δ-9-THC and the synthetic CB1R agonist WIN55212-2 reduce high IOP in patients with glaucoma [50,51]. These modulatory effects of cannabinoids in IOP, however, are mediated by CB1 receptors localised to non-neural tissues in the anterior part of the eye which regulate aqueous humor production (see [40,52] for reviews). Although topical application of cannabinoids to treat eye diseases presents the challenge of solubilizing highly lipophilic molecules, successful delivery of eCBs and cannabinoid drugs to the anterior segment of the eye has been achieved with a subsequent reduction of IOP [53,54]. A strategy to locally deliver drugs that modulate the eCB system in the retina for neuroprotection, however, remains a challenge.

Despite the neuroprotective potential of eCBs in the treatment of some degenerative retinal diseases, understanding how visual function is affected by changes in the retinal eCB system function due to pharmacological manipulation as well as in recreational cannabis users is crucial. Regular cannabis use was shown to alter bipolar and RGC function in humans evidenced by pattern electroretinogram recordings [55,56]. Furthermore, regular cannabis use alters the steady state visual evoked potential measured in the visual cortex [57] and the responses to low spatial frequencies, suggesting an impairment of the magnocellular pathway [58]. Moreover, cannabis consumption was reported to reduce visual acuity, stereoacuity and contrast sensitivity together with an impairment in self-perceived visual quality related to halo perception and night-vision [59]. At the behavioral level, acute cannabis smoking by occasional users was associated with a decline in driving performance as assessed by using a car-based driving simulator [60,61] and by an impairment in actual driving performance in a dose-dependent manner [62].

## 5. Conclusions

Our results provide evidence that supports a role of tonic endocannabinoids in various aspects of retinal processing such as modulation of response strength to visual stimulation, receptive field organization and contrast sensitivity. Interestingly, our results revealed a paradoxical effect of anandamide at the synaptic and at the RGC level: while FAAH inhibition reduced the amplitude of visually evoked post-synaptic potentials, surprisingly it enhanced RGC excitability. Thus, high levels of anandamide in the retina amplify the signals that propagate to higher visual areas and therefore, vision becomes sensitised. Whether or not this enhancing effect is mediated via CBRs or other non-CBR targets such as TRPV1 channels remains to be clarified. A more thorough understanding of the retinal eCB system and its signalling pathways will shed light on the effects of drugs that act on different components of the cannabinergic system.

## Data Availability

The data are available upon reasonable request to the corresponding author.

## Supplementary Materials

The following supporting information can be downloaded at: https://www.mdpi.com/article/10.3390/ijerph191912460/s1, Figure S1: Schematic diagram of the recording set up. Retinal tissue was mounted photoreceptor side down in a recording chamber under continuous perfusion with Ames medium. Ganglion cells were visualized under infrared illumination on a monitor using a digital camera controlled via a computer. The computer was also connected to a digitizer the controlled and received signals from the patch-clamp amplifier. The output of a monochromatic OLED was passed via a lens system and combined with infrared illumination with a beamsplitter to project stimuli to the retina via a lens system from below via the microscope optics (condenser) and allow visualization of the tissue The inlet line was also used to perfuse Ames medium + URB597.

## Abbreviations

2-AG	2-acylglycerol
AEA	anandamide
C_50_	half saturation point (contrast at 50% of maximum LE-PSP amplitude)
CB1R	cannabinoid receptor type 1
CB2R	cannabinoid receptor type 2
CBD	cannabidiol
CNS	central nervous system
DMSO	dimethyl sulfoxide
DoG	difference-of-Gaussian
eCB	endocannabinoid
ERG	electroretinogram
FAAH	fatty acid amide hydrolase
IOP	intraocular pressure
IPL	inner plexiform layer
LE-PSP	light-evoked postsynaptic potential
NKCC1	Na^+^-K^+^-2Cl^−^ co-transporter 1
OLED	organic light-emitting diode
OPL	outer plexiform layer
RGCs	retinal ganglion cells
SI	suppression index
THC	∆9-tetrahydrocannabinol
TRPV1	transient receptor potential vanilloid type 1
