# Supplementary material for: Tonic Endocannabinoid Levels Modulate Retinal Signaling
Source: Int J Environ Res Public Health. 2022 Sep 30;19(19):12460. doi: 10.3390/ijerph191912460 (PMC9566182; doi:10.3390/ijerph191912460)
Supplement: Supplementary file 1 [file ijerph-19-12460-s001.zip › ijerph-1850329-supplementary.pdf]

Supplementary Materials:

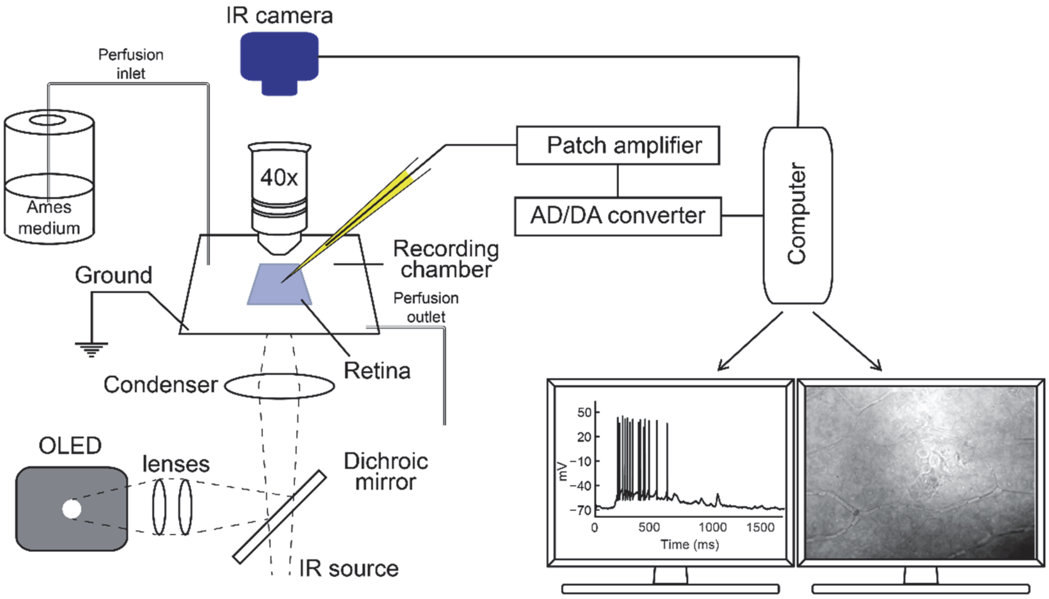

Supplementary Figure S1. Schematic diagram of the recording set up. Retinal tissue was mounted photoreceptor side down in a recording chamber under continuous perfusion with Ames medium. Ganglion cells were visualized under infrared illumination on a monitor using a digital camera controlled via a computer. The computer was also connected to a digitizer the controlled and received signals from the patch-clamp amplifier. The output of a monochromatic OLED was passed via a lens system and combined with infrared illumination with a beamsplitter to project stimuli to the retina via a lens system from below via the microscope optics (condenser) and allow visualization of the tissue The inlet line was also used to perfuse Ames medium + URB597.
